# Graphene Nanoplatelets Reinforced ABS Nanocomposite Films by Sonication-Assisted Cast Film Technique for Emission Shielding Application

**DOI:** 10.3390/ma18112645

**Published:** 2025-06-05

**Authors:** Mohammed Iqbal Shueb, Noraiham Mohamad, Syarfa Zahirah Sapuan, Yee See Khee, Dewi Suriyani Che Halin, Andrei Victor Sandu, Petrica Vizureanu

**Affiliations:** 1 Polymer Processing and Prototyping Development Group, Radiation Processing Technology Division, Malaysian Nuclear Agency, Kajang 43000, Selangor, Malaysia; 2 Fakulti Teknologi dan Kejuruteraan Industri dan Pembuatan, Universiti Teknikal Malaysia Melaka, Hang Tuah Jaya, Durian Tunggal 76100, Melaka, Malaysia; noraiham@utem.edu.my; 3 Faculty of Electrical and Electronic Engineering, Universiti Tun Hussein Onn Malaysia, Parit Raja, Batu Pahat 86400, Johor, Malaysia; syarfa@uthm.edu.my (S.Z.S.); skyee@uthm.edu.my (Y.S.K.); 4 Faculty of Chemical Engineering & Technology, Universiti Malaysia Perlis (UniMAP), Kompleks Pusat Pengajian Jejawi 2, Taman Muhibbah, Arau 02600, Perlis, Malaysia; dewisuriyani@unimap.edu.my; 5 Centre of Excellent Geopolymer and Green Technology (CeGeoGTech), Universiti Malaysia Perlis (UniMAP), Kompleks Pusat Pengajian Jejawi 2, Taman Muhibbah, Arau 02600, Perlis, Malaysia; 6 Faculty of Materials Science and Engineering, “Gheorghe Asachi” Technical University of Iasi, 67 Prof. D. Mangeron Blvd., 700050 Iasi, Romania; petrica.vizureanu@academic.tuiasi.ro; 7 Romanian Inventors Forum, Str. Sf. P. Movila 3, 700089 Iasi, Romania; 8 Academy of Romanian Scientists, 54 Splaiul Independentei St., Sect. 5, 050094 Bucharest, Romania

**Keywords:** ABS, GNP, wettability, thermal stability, dielectric strength, EMI shielding effectiveness, ultraviolet absorption

## Abstract

The rapid proliferation of electronic devices has heightened the demand for efficient electromagnetic interference (EMI) shielding materials, as conventional alternatives increasingly fall short in mitigating harmful electromagnetic radiation. In this study, we report the fabrication of acrylonitrile butadiene styrene (ABS) nanocomposite films reinforced with graphene nanoplatelets (GNPs), offering a promising solution to this growing challenge. A persistent issue in incorporating GNPs into the ABS matrix is their poor wettability, which impedes uniform dispersion. To overcome this, a sonication-assisted casting technique was employed, enabling effective integration of GNPs at loadings of 1, 3, and 5 wt%. The resulting nanocomposite films exhibit uniform dispersion and enhanced functional properties. Comprehensive characterization using FESEM, UV-Vis spectroscopy, TGA, DSC, FTIR, and dielectric/EMI analyses revealed significant improvements in thermal stability, UV absorption, and dielectric behavior. Notably, the films demonstrated moderate EMI shielding effectiveness, reaching 0.0064 dB at 4 MHz. These findings position the developed GNP-reinforced ABS nanocomposites as promising candidates for advanced applications in the automotive, aerospace, and electronics industries.

## 1. Introduction

The incorporation of carbon-based nanomaterials into polymer matrices has attracted extensive interest due to their exceptional electrical, thermal, and mechanical properties, which are highly beneficial in the fabrication of high-performance nanocomposites [1,2]. Acrylonitrile butadiene styrene (ABS), a widely used engineering thermoplastic, is particularly valued for its good processability, chemical resistance, and mechanical strength. However, limitations remain in enhancing its functional properties, such as water repellency, thermal stability, and electromagnetic interference (EMI) shielding [1,2]. Recent developments in graphene-based nanofillers have shown significant potential in overcoming these limitations, especially through the integration of graphene nanoplatelets (GNPs) [2,3,4].

GNPs, owing to their two-dimensional geometry, high aspect ratio, and large surface area, offer unique advantages for tailoring the structural and functional properties of polymer matrices. When embedded into ABS, GNPs can effectively improve mechanical strength, stiffness, and EMI shielding while maintaining processability [3,4]. The enhancements also extend to wettability and thermal properties, where even low GNP loadings have demonstrated significant gains [5,6,7]. These improvements are due to the formation of conductive and thermal pathways that restrict polymer chain mobility and enhance interfacial interactions, resulting in increased thermal stability and resistance to degradation [6,7].

Beyond mechanical and thermal benefits, GNPs have also shown promise in modulating surface energy and wettability, which are vital for applications in coatings, adhesives, and biomedical devices [8,9,10,11,12,13,14,15]. The wettability of nanocomposites can be assessed via contact angle measurements and surface energy estimations, which reveal critical insights into interfacial behavior. Additionally, optical properties such as energy bandgap and carbon cluster distribution have gained increasing attention for sensor and photovoltaic applications [10]. Incorporating GNPs into polymer matrices leads to the formation of conductive networks, enhancing dielectric and EMI shielding functionalities while opening avenues for optical and photonic uses [10].

Nevertheless, several challenges hinder the full exploitation of GNP-based ABS nanocomposites. These include dispersion difficulties due to the incompatibility between hydrophobic GNPs and the polar segments of ABS, often resulting in agglomeration at higher loadings. Traditional methods like melt blending and in situ polymerization are limited in overcoming this issue. To address this, innovative techniques such as sonication-assisted dispersion are being employed [9,11]. This approach enhances nanoplatelet exfoliation and distribution by inducing cavitation, reducing thermal degradation, and improving film uniformity even at low loadings [11].

In addition to physical and thermal improvements, synergistic effects between electrical, dielectric, and optical properties are also gaining relevance. However, their interplay remains underexplored. While functionalization techniques—such as introducing hydroxyl, carboxyl, or amine groups—can improve compatibility, this study isolates the effects of pristine GNPs dispersed via sonication to focus on physical interactions.

Moreover, sustainable material design is gaining traction across polymer nanocomposite research. Waste-derived and environmentally friendly nanofillers have been shown to provide scalable and eco-conscious solutions [16], and fabrication strategies including surface functionalization, controlled dispersion, and matrix-filler compatibility optimization are critical for multifunctionality [17]. Sonication-assisted techniques, in particular, have demonstrated effectiveness in thin-film nanocomposites by ensuring interfacial bonding and structural uniformity [18].

Despite the proven benefits of GNP-filled composites [2,3,6,7,10], important research gaps persist, particularly in understanding the coupled effects of wettability, dielectric behavior, thermal stability, and optical properties. This study aims to fill these gaps by utilizing a sonication-assisted casting film method to fabricate GNP-reinforced ABS nanocomposites. By systematically evaluating their structural, thermal, dielectric, EMI shielding, and optical characteristics, the work contributes to the broader advancement of sustainable, multifunctional nanocomposites for high-performance applications across various industries.

## 2. Materials

In this study, we utilized Graphene nanoplatelets (GNPs) with a thickness <100 nm and lateral dimensions ranging from 1 to 50 μm were obtained from The Sixth Element (Changzhou) Materials Technology Co., Ltd. (Changzhou, China). Acrylonitrile butadiene styrene (ABS) pellets (TOYO, Penang, Malaysia) and analytical grade acetone (Merck, Darmstadt, Germany) were supplied by Manshurin Drive Sendirian Berhad (Bangi, Malaysia). All materials were used as received without further purification.

### 2.1. ABS/GNPs Film Nanocomposite Fabrication

**Figure 1:**
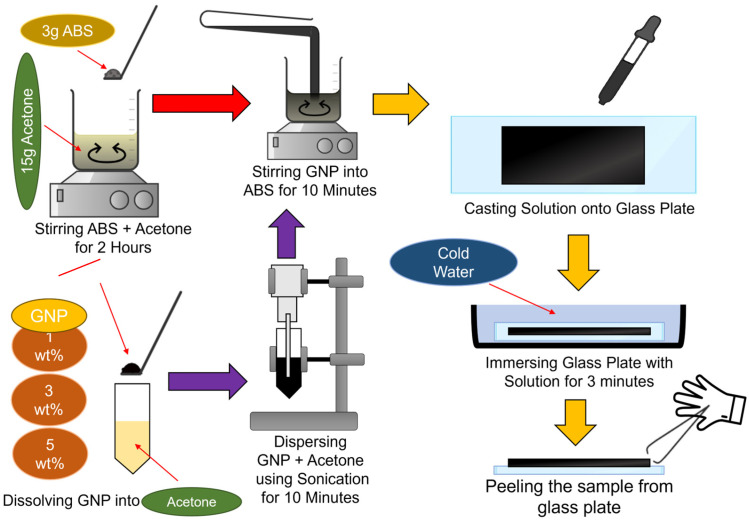
Illustration of sonication-assisted casting film technique of GNP-reinforced ABS nanocomposite films.

**Figure 2:**
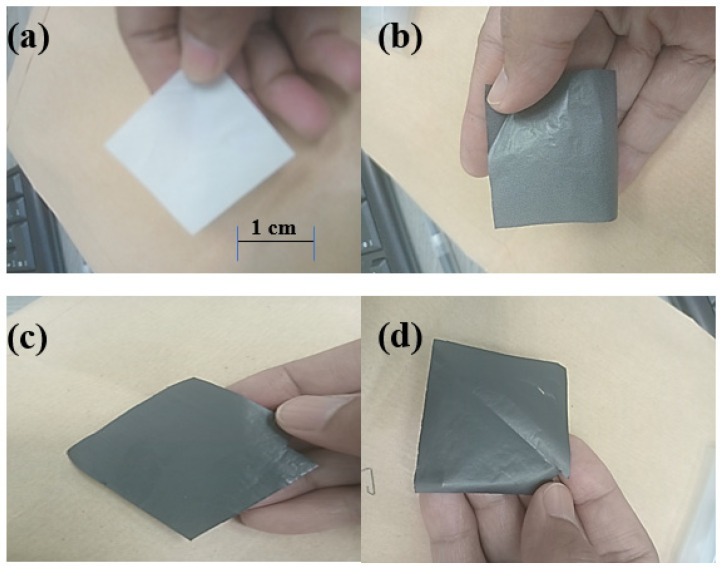
The ABS-GNP nanocomposite film at various GNPs loading of (**a**) without, (**b**) 1 wt%, (**c**) 3 wt% and (**d**) 5 wt% of GNPs loading.

Figure 1 shows the illustration of a sonication-assisted casting film technique set-up. Three different formulations of GNP-reinforced ABS nanocomposite films were investigated (Table 1). The range of 1–5 wt% GNP applied is to maintain dispersion stability and minimize agglomeration, which compromises mechanical and electrical properties. Higher loading (>5 wt%) in preliminary trials led to phase separation and brittleness. This is consistent with literature targeting optimal GNP loadings for percolation and dielectric enhancement without aggregation [2]. For the film preparation, a solution was prepared by dissolving 3 g of ABS granules in 15 g of acetone and agitating the solution at ambient temperature for two hours. Subsequently, GNP was incorporated into the solution at concentrations of 1%, 3%, or 5% by weight. For each GNP loading, three films were fabricated, and three measurements per sample were taken for FTIR, DSC, TGA, dielectric, and SE tests to ensure repeatability. A continuous sonication was applied to the blend for 10 min to achieve uniformity. To facilitate the removal of the dark solutions, they were poured into a glass petri dish and submerged in cool water for 2–3 min. Then, the films (Figure 2) were subjected to various testing and materials characterization. All films maintained a consistent thickness of approximately 0.2 mm, measured using a digital micrometer, to ensure uniform dielectric and EMI shielding measurements across all GNP loadings.

### 2.2. The Contact Angle and Surface Energy

The wettability of a nanocomposite can be determined via a contact angle and surface energy methodology. It was conducted in two steps: (1) contact angle measurement and (2) surface energy (SFE) calculation before the data can be interpreted. The first step was carried out using a contact angle goniometer at room temperature by placing a drop of water on the surface of the nanocomposite film. Then, an SFE calculation was performed by analyzing the contact angle data. The Owens-Wendt-Rabel-Kaelble (OWRK) approach was used in this step. Then, the data on contact angle and SFE were used to evaluate the wettability of the nanocomposite films at different GNP loadings. Larger contact angle values (>90°) indicate poor wettability, while smaller contact angle values (<90°) indicate good wettability [19].

### 2.3. TGA Analyzer

The thermogravimetric analysis (TGA) was carried out using a Perkin Elmer (Waltham, MA, USA) Pyrist 6 TGA analyzer. Samples (~5 mg) were placed in a platinum pan, and the experiments were conducted in nitrogen at a 60 mL/min flow rate. Next, samples were scanned from 30 °C to 800 °C at a heating rate of 10 °C min.

### 2.4. DSC Analyzer

Thermal characteristics of the nanocomposite samples were investigated under nitrogen using DSC7 device (Perkin Elmer, Waltham, MA, USA). The samples (approximately 10 mg) were heated and cooled at 10 K min^−1^ from 25 °C to 300 °C. The curves were analyzed to determine the glass transition temperature (T_g_) and melting behaviour.

### 2.5. FTIR Spectroscopy

**Figure 3:**
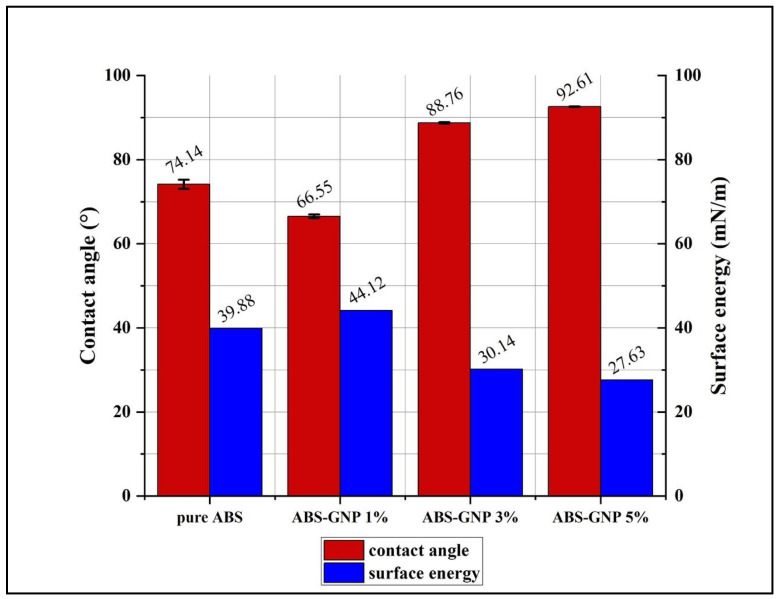
Relationship between contact angle and surface energy.

Fourier-transform infrared (FTIR) spectra were obtained at a resolution of 4.0 cm^−1^ in the wavenumber range of 4000–400 cm^−1^ using an FTIR spectrophotometer (Bruker, Ettlingen, Germany) as shown in Figure 3. 10 at 25 °C to investigate the variation in the surface structure of the GNPs in ABS polymer. FTIR analyses were performed using the conventional KBr pellet technique.

### 2.6. Dielectric Test

In this study, the dielectric properties of the thin film were characterized using the Keysight 16451B (Keysight Technologies, Inc., Santa Rosa, CA, USA) dielectric test fixture in conjunction with a Keysight impedance analyzer following ASTM D150 standards [8] During the measurement process, the material under test is positioned between two electrodes of the test fixture, forming a capacitor. Subsequently, the impedance analyzer is employed to determine the capacitance generated by the fixture and convert it to the material’s complex permittivity. The Keysight N1500A Material Measurement Suite facilitates the conversion procedure. It is important to note that the test fixture requires the thickness of the material under test to be less than 10 mm, with a diameter falling within the range of 10–56 mm.

The effect of adding GNP into ABS matrices on thermal conductivity could be measured using Equation (1) [8], where σ denotes the conductivity, ω represents the angular frequency, and ε_o_ signifies the permittivity of free space (8.854 × 10^−^^12^).

σ = ε″·ώε_0_ (1)

### 2.7. EMI Shielding Effectiveness (SE)

Shielding effectiveness (SE), it is represented as the logarithm of ratio of incident field to transmitted field strength in decibel (dB) as shown in Equation (2), where E, H and P represent the electric field, magnetic field and power density of the EM wave respectively, the subscript i and t represent the incident and transmitted component.

SE = 20 log(Ei/Et) = 20 log (Hi/Ht) = 20 log (Pi/Pt)(2)

According to Schelkunoff’s Theory (2), the shielding mechanism of a material is contributed by three types of losses (in dB), namely reflection loss (R), absorption loss (A), and multiple re-reflection loss (M) as shown in Equation (3). As electromagnetic waves (EMWs) near the surface of shielding materials, some of them are reflected because of impedance mismatch. Subsequently, the remaining waves penetrate the material interface, where their intensity decreases exponentially. Upon reaching the opposite surface of the shielding materials, a fraction of the waves undergoes re-reflection (multiple-internal reflection), while the remainder continues to transmit through.

SE (dB) = R + A + M(3)

The formulation of reflection loss, absorption loss and multiple re-reflection loss are listed in (4), (6), and (8). η signifies the characteristic impedance of the shielding material, calculable through Equation (5). Here, μ, σ, and ε denote the permeability, conductivity, and real part of permittivity (dielectric constant) of the material, respectively. γ represent the propagation constant of the electromagnetic wave within the material.

R_dB_ = 20 log_10_ [(η_o_ + η)^2^/(4η_o_ η)] (4)

η_o_ = [ √(jωμ/(σ + jωε)] (5)

A_dB_ = 20 log_10_ (e^γt^) (6)

γ = √(jωμ(σ + jωε))(7)

M = 20 log_10_ [1 − ((η_o_ − η)/(η_o_ + η))^2^ e^(−2γt)^] (8)

### 2.8. Field Emission Scanning Electron Microscope (FESEM)

In this study, we characterized the particle size and surface morphology using a field emission scanning electron microscope (FESEM), specifically the JEOL JSM-7600F model from Japan (Jeol Ltd., Tokyo, Japan). This microscope is equipped with an energy dispersive X-ray spectrometer (EDS), the OXFORD X-MAX Energy 200 Premium from the UK (Oxford Instruments, Abingdon, UK), and a silicon drift detector with an active area of 80 mm^2^. The samples were prepared by adhering them to a stub using double-sided adhesive tape. The samples were coated with the thinnest possible layer of gold or platinum before being placed in the FESEM chamber. The secondary electron imaging mode was used with an accelerating voltage of 5 kV. The samples were carefully loaded into a specialized FESEM sample holder and then introduced into the FESEM chamber, which was maintained at a vacuum of approximately 1.0 × 10^−4^ Pa. The surface morphology images were scanned using an electron beam with an approximate voltage of 2.00 kV, a detector mode of LEI SEM, and a large scan distance (WD) of about 9.6–9.8 mm. The optimal magnification range to obtain the best images was between 25,000× and 50,000×. This analysis evaluated the efficiency of sonication-assisted cast film techniques in improving interaction between graphene nanoplatelets and the ABS matrix.

## 3. Results and Discussion

### 3.1. Optical Contact Angle (OCA)

**Figure 4:**
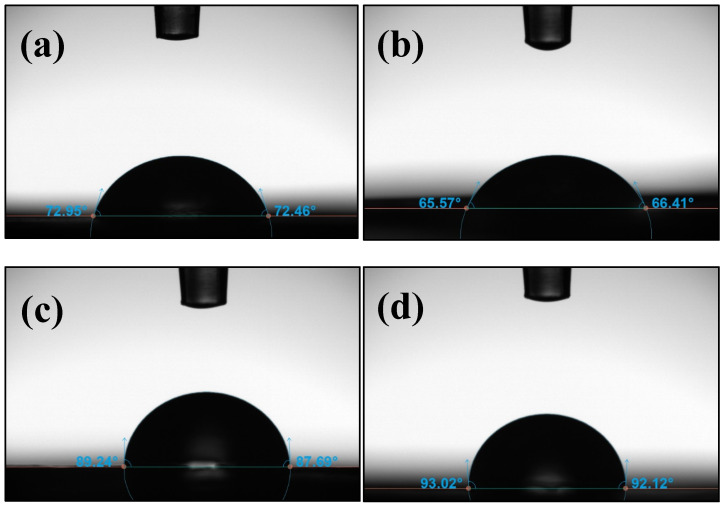
The water contact angle images of polymer nanocomposites of (**a**) pure ABS, (**b**) ABS-GNP 1 wt%, (**c**) ABS-GNP 3 wt%, and (**d**) ABS-GNP 5 wt%.

The wettability and surface energy of the ABS-GNP polymer nanocomposite were evaluated using the optical contact angle (OCA) method. Figure 3 and Figure 4 and Table 2 illustrate the correlation between the contact angle and surface energy and the water contact angle of pure ABS and its polymer nanocomposites, respectively.

Figure 3 and Table 2 display the relationship between contact angle and surface energy and the summary of water contact angle and surface energy of ABS and its nanocomposites, respectively. Pure ABS Polymer (0% GNPs) demonstrates a hydrophilic surface characteristic, indicating a preference for water, as indicated by the contact angle of 74.14 degrees. This is likely due to polar groups within the ABS polymer, which enable it to interact with water molecules [20]. As an effect of incorporating 1% GNPs, the contact angle was reduced to roughly 66.54 degrees. The observed decrease suggests that the hydrophobicity of the surface is progressively increasing when compared to ABS in its unmodified state. The hydrophobic properties of GNPs have been extensively acknowledged, and even trace amounts of these particles have the ability to alter the surface characteristics of the composite material. The increased hydrophobicity is a result of the GNPs’ inherent ability to absorb water molecules.

The incorporation of graphene nanoplatelets (GNPs) into acrylonitrile butadiene styrene (ABS) films significantly influenced their surface wettability and energy characteristics. As shown in Figure 3 and Figure 4 and Table 2, the contact angle values increased markedly with the addition of 3 wt% (88.76°) and 5 wt% (92.61°) GNPs, indicating a distinct enhancement in surface hydrophobicity compared to both the pure ABS matrix and the composite containing 1 wt% (66.54°) GNPs. These findings suggest that increasing GNP content alters the surface properties of the composites, promoting a transition from hydrophilic to hydrophobic behavior.

This shift in wettability can be primarily attributed to the increased surface coverage by GNPs at higher loadings. The graphene nanoplatelets, being inherently hydrophobic, form a discontinuous but extensive surface layer that effectively shields the more hydrophilic ABS substrate from direct contact with water. As the GNP concentration increases, this surface masking effect becomes more pronounced, leading to higher contact angles. This observation aligns with previous studies reporting similar hydrophobic behavior in graphene- and carbon-based polymer composites [20,21].

Additionally, the presence of GNPs may alter the interfacial energy between the composite surface and water, contributing to reduced wettability. At elevated concentrations, GNPs can increase the interfacial tension by creating thermodynamically unfavorable interactions between water molecules and the composite surface. The hydrophobic domains formed by GNPs hinder water spreading and penetration, further enhancing water repellency.

A secondary mechanism contributing to this behavior is the barrier effect imparted by GNPs. Due to their high aspect ratio and planar morphology, GNPs can form tortuous diffusion paths within the polymer matrix, effectively restricting water molecule penetration. This phenomenon has been well-documented in polymer nanocomposite systems and supports the hypothesis that GNPs increase the material’s resistance to surface hydration [22].

Moreover, the possibility of GNP aggregation at higher loadings introduces nanoscale surface features that influence the wetting regime. SEM micrographs show increased surface roughness and discontinuities that may trap air pockets, promoting a Cassie–Baxter-type wetting behavior. This air entrapment reduces the contact area between water and the composite surface, which contributes to the elevated contact angle values and apparent hydrophobicity [23].

In contrast, the composite containing 1 wt% GNPs demonstrated a moderate increase in surface energy, suggesting different surface interactions at lower filler concentrations. At this loading, the GNPs were more uniformly dispersed within the ABS matrix, enabling enhanced polymer-filler interfacial interactions. This uniform distribution may modify the surface chemistry or topography, increasing the polar and dispersive components of surface energy. Furthermore, GNPs with residual oxygen-containing functional groups or edge defects could introduce polar sites capable of hydrogen bonding, thereby raising the overall surface energy [24,25,26].

Interestingly, despite the increase in hydrophobicity at 3 wt% and 5 wt% GNP concentrations, a notable decrease in surface energy was observed. This apparent contradiction can be explained by the dominant effect of GNP surface coverage and aggregation, which lowers the surface free energy by masking the more polar ABS substrate. The microstructural changes induced by higher GNP content, such as increased crystallinity or chain alignment within the ABS matrix, may also reduce the surface energy [27]. Additionally, strong interfacial interactions between aggregated GNP domains and ABS chains may contribute to a more cohesive surface with lower surface energy [28].

The increased surface roughness detected by SEM further influences wettability but may not proportionally raise the surface energy in terms of chemical affinity. Instead, the roughness creates a composite interface of solid and trapped air pockets that lowers the effective surface energy experienced by contacting liquids, consistent with Cassie–Baxter wetting behavior [29].

Overall, these results demonstrate that GNP addition leads to a concentration-dependent modulation of both surface wettability and energy. While the increased GNP content enhances hydrophobicity through surface coverage, interfacial tension, and structural roughness, it simultaneously reduces surface energy by masking polar ABS regions and inducing microstructural modifications. Further investigations using techniques such as atomic force microscopy (AFM), X-ray photoelectron spectroscopy (XPS), and surface energy component analysis would be valuable for gaining deeper insight into the physicochemical mechanisms underlying these observations.

### 3.2. Thermogravimetric Analysis (TGA)

**Figure 5:**
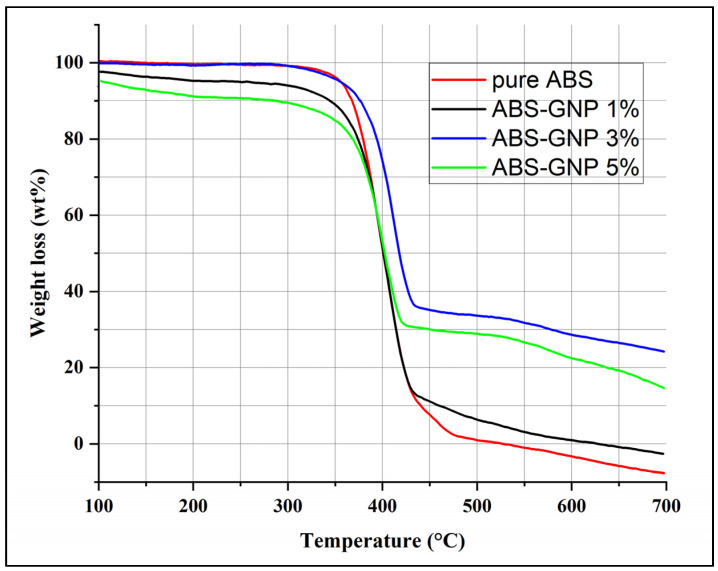
TGA curves of ABS-GNP polymer nanocomposites.

The thermal degradation stability of Acrylonitrile Butadiene Styrene (ABS) composite films containing varying concentrations of graphene nanoplatelets (GNPs) (1 wt%, 3 wt%, and 5 wt% nanocomposites) was investigated using thermogravimetric analysis (TGA) under a heating rate of 10 °C/min in an inert atmosphere (Figure 5). The TGA outcomes of ABS and its nanocomposites are depicted in Figure 5, whereas a summary of the weight loss values at different temperatures can be found in Table 3. The comparative thermal stability of the nanocomposites and purified ABS is substantiated by the results obtained from the data analysis. T_20_, T_50_, and T_80_ represent the temperatures at which decomposition takes place, respectively [25].

The respective rates of weight loss at these temperatures are 20%, 50%, and 80%. The maximum recorded temperature increases of 372.51 °C, 407.51 °C, and 432.51 °C were found in nanocomposites comprising 3% GNPs, in contrast to those containing 1% and 5% GNPs, respectively. An increase in temperature of around 20 °C, 5 °C, and 2 °C was detected at T_20_, T_50_, and T_80_, respectively, when nanocomposites contained 3%wt%. Several factors contribute to the improved thermal stability of nanocomposites incorporating graphene nanoplatelets (GNPs) as opposed to pure ABS (Acrylonitrile Butadiene Styrene). These factors encompass the distinctive characteristics of the GNPs as well as their interaction with the polymer matrix: (a) Graphene nanoplatelets exhibit remarkable thermal conductivity as a result of their two-dimensional configuration and carbon atoms that are sp^2^ hybridized. GNPs have been observed to efficiently disperse heat away from the polymer chains when incorporated into the polymer matrix; this results in a decrease in localized heating and a postponement of thermal degradation. (b) Barrier Effect: By integrating GNPs into the polymer matrix, a physical barrier can be established, impeding the diffusion of volatile decomposition products that are generated during the process of thermal degradation. The degradation process is slowed by this barrier effect, which contributes to enhanced thermal stability. (c) Improved Dispersion: Ensuring the appropriate distribution of GNPs throughout the polymer matrix is critical in order to optimize their efficiency in augmenting thermal stability. A greater interfacial area is created between the GNPs and the polymer matrix due to good dispersion; this increases the efficiency of heat transfer and barrier effects. (d) Chemical Interaction: By functionalizing GNPs, interactions with the polymer matrix can be enhanced, resulting in improved phase compatibility and adhesion. The incorporation of graphene nanoplatelets (GNPs) significantly enhances the thermal stability of ABS/GNP nanocomposites through several interrelated mechanisms. GNPs act as nucleating agents, promoting a more ordered crystalline structure within the polymer matrix. This increased crystallinity reduces molecular mobility and raises the activation energy required for thermal degradation, thereby contributing to improved thermal resistance [25,26].

Moreover, strong interfacial interactions between GNPs and the ABS matrix contribute to the mechanical integrity of the composite, which in turn mitigates the onset of thermal degradation under physical stress [25]. The high aspect ratio and large surface area of GNPs introduce a pronounced barrier effect, restricting the diffusion of volatile decomposition products during heating and thus slowing the degradation rate [26].

The inherently high thermal conductivity of GNPs further facilitates efficient heat dissipation throughout the composite, reducing localized heating and minimizing thermal stress on polymer chains [27,28,29]. These thermal management properties are instrumental in delaying the onset of decomposition.

Additionally, the synergy between GNPs and flame-retardant or stabilizing additives can further optimize thermal performance. In particular, GNPs may promote the in situ formation of graphene oxide (GO) during degradation, due to oxygen-containing functional groups on their surfaces. The resulting GO layer can act as a physical barrier, protecting the underlying polymer matrix from oxidative and thermal damage [30,31,32].

Taken together, these findings support the conclusion that the enhanced thermal stability observed in ABS/GNP nanocomposites arises from a multifaceted interplay of nucleating effects, barrier mechanisms, improved interfacial interactions, and effective thermal dissipation, as consistently reported in the literature [25,26].

### 3.3. Differential Scanning Calorimetry (DSC)

**Figure 6:**
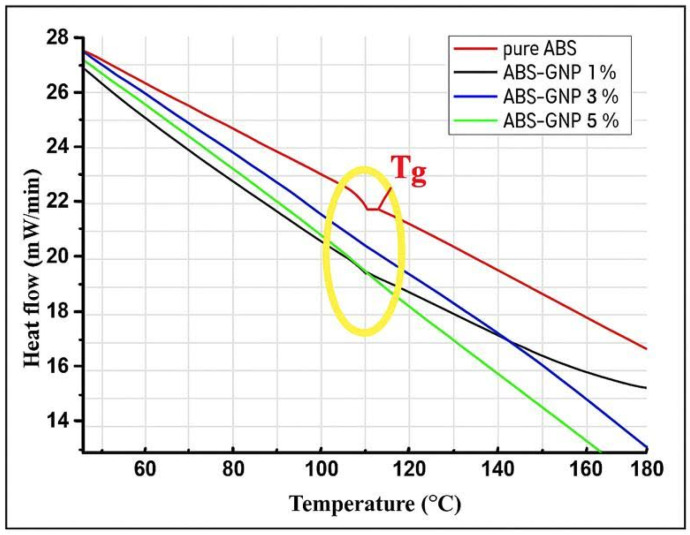
DSC curves of pure ABS and ABS-GNPs nanocomposites. The glass transition temperature (T_g_) of the sample was highlighted using yellow colored oval shape on the TGA graph to clearly mark the onset of polymer chain mobility changes.

The glass transition temperature (T_g_) is a critical quantity in the field of polymer research. It signifies the temperature at which an amorphous polymer undergoes a transition from a stiff and glass-like state to a more pliable and rubbery state. The addition of nanoparticles, specifically graphene nanoplatelets (GNPs), to polymer matrices like Acrylonitrile Butadiene Styrene (ABS), can have a substantial impact on the thermal characteristics of the resulting nanocomposites. The T_g_ of many ABS-GNP nanocomposites was determined to fall within the range of 109–125 °C. This finding indicates that including GNPs as a filler has appreciable influence on the thermal properties of the ABS matrix. The data in Figure 6 and Table 4 indicate that pure ABS exhibits a T_g_ peak at 109.82 °C, the lowest among all the samples studied. In contrast, all the GNP-reinforced ABS nanocomposites demonstrated higher T_g_ values compared to pure ABS, highlighting the effect of graphene nanoplatelet incorporation on improving the thermal properties of the material. The observed increase in glass transition temperature (T_g_) aligns with the typical behavior of polymer nanocomposites, wherein the incorporation of graphene enhances intermolecular interactions, thereby improving thermal stability. Notably, nanocomposites containing 5 wt% graphene nanoplatelets (GNPs) exhibit the highest T_g_ at 123.81 °C, surpassing those with 1 wt% and 3 wt% GNPs. The slight decrease in T_g_ at 3 wt% may arise from localized GNP agglomeration affecting chain mobility. This aligns with FESEM evidence of early-stage clustering at intermediate loading, reducing confinement effects compared to 1% [32]. An association can be detected between the thermal characteristics of the nanocomposite and the concentration of GNPs. It is possible that the interaction between the filler and the polymer matrix may not be sufficient to provide a statistically significant change in T_g_ at lower concentrations of GNP, such as 1%. On the other hand, a rise in GNP concentration enhances the strengthening effects, resulting in a gradual increase in T_g_ over a period of time. The observed range of T_g_ values in nanocomposites containing 1%, 3%, and 5% graphene nanoplatelets (GNPs) indicates that the observed variation aligns with this trend.

Moreover, the rise in T_g_ resulting from the inclusion of GNPs can be ascribed to various factors: (a) Augmented interfacial contacts: The inclusion of GNPs in the polymer matrix can result in intensified interactions between the nanoparticles and the polymer chains. This phenomenon can limit the movement of polymer chains, resulting in an elevation of the glass transition temperature (T_g_). (b) Enhanced stiffness: Graphene Nanoplatelets (GNPs), due to their high rigidity, can serve as physical obstacles inside the polymer structure, impeding molecular movement and thereby elevating the glass transition temperature. (c) Uniform dispersion: Ensuring the proper distribution of GNPs within the polymer matrix is essential for optimizing their impact on the glass transition temperature (T_g_). When evenly distributed, Graphene Nanoplates (GNPs) can efficiently strengthen the polymer matrix, leading to an elevation in the T_g_. (d) The confinement effect refers to the restriction of polymer chains near GNPs, which might alter the movement of the chains and consequently impact the T_g_ of the nanocomposite. To summarise, the rise in T_g_ seen in ABS-GNP nanocomposites with increasing GNP content can be due to a combination of variables such as improved interfacial contacts, heightened stiffness, uniform dispersion, and confinement effects [33,34,35,36].

### 3.4. Fourier Transform Infrared Analysis (FTIR)

Fourier Transform Infrared Spectroscopy (FTIR) was used to explore the chemical interaction and stability of Acrylonitrile Butadiene Styrene (ABS) and graphene nanoplates (GNPs) nanofiller.

**Figure 7:**
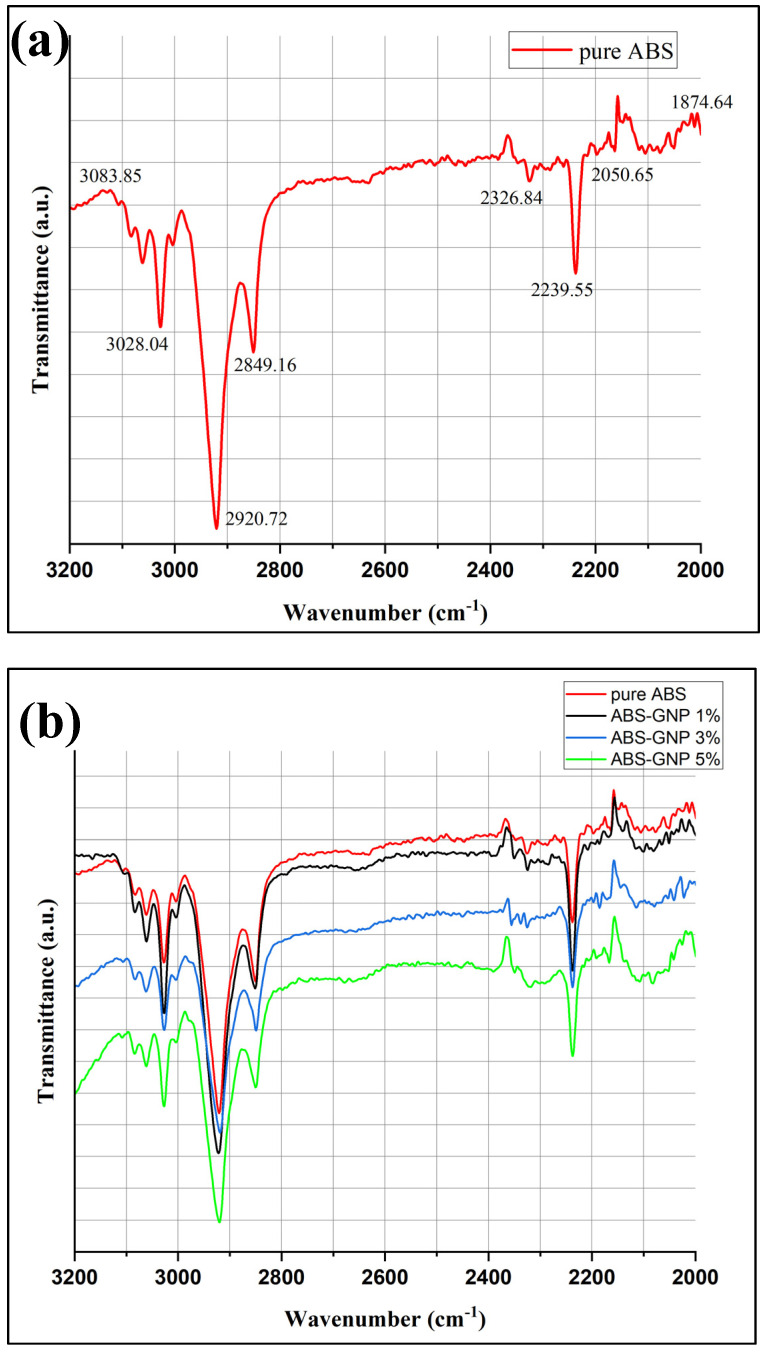
(**a**) FTIR spectra of pure ABS (**b**) FTIR spectra of ABS/GNPs nanocomposites at different loading.

Figure 7a,b displays the FTIR spectra of pure ABS and nanocomposites with varying percentages of GNPs respectively. The addition of graphene nanoplatelets (GNPs) to an acrylonitrile butadiene styrene (ABS) matrix in nanocomposites leads to a shift towards longer wavelengths in the position of band intensities, particularly in the range of 3100–3000 cm^−1^. Additionally, the strength of the C=O band around 2920 cm^−1^ is reduced. These changes can be attributed to various mechanisms. Matrix representing the initial GNPs and Aggregate Demand (ABS) The observed redshift in peak positions may be attributed to the interactions between the ABS matrix and the GNPs. The introduction of GNPs into the matrix can lead to variations in the vibrational frequencies detected in the Fourier Transform Infrared (FTIR) spectra. These variations may arise from the presence of new molecular environments and changes to the local molecule vibrations. π-π stacking interactions between the conjugated structure of graphene and the polymer chains of ABS have the potential to affect the vibrational modes of the polymer backbone [37]. Changes in the spatial arrangement of molecules that occur in close proximity to one other. Incorporating GNPs into ABS polymer chains can lead to changes in their conformation. This may lead to variations in the vibrational frequencies of specific functional groups within the polymer matrix. Furthermore, it can also influence the allocation of molecular energy levels. Hence, changes in the position and intensity of Fourier Transform Infrared (FTIR) peaks occur [38]. The π-electrons that are not confined to a specific location contribute to the increased electron density that is observed in GNPs. The introduction of GNPs may lead to charge transfer interactions between the ABS matrix and the nanoparticles. Changes in FTIR spectra can occur due to these interactions, which modify the electrical structure of the polymer and thus impact its vibrational modes [39]. The FTIR spectra can be significantly affected by the dispersion and aggregation of GNPs within the polymer matrix. The agglomeration or aggregation of GNPs has the potential to alter the immediate environment around the polymer chains, leading to changes in vibrational frequencies. The interactions between GNPs and the polymer matrix may be enhanced when they are dispersed appropriately, which can impact the observed spectral characteristics [40]. The progressive drop in the strength of the C=O band at 2920 cm^−1^ may be caused by changes in the chemical environment surrounding carbonyl groups in the ABS matrix. The presence of GNPs can reduce the strength of the infrared absorption of C=O bonds by obstructing or protecting them. The cause may be attributed to changes in molecular packing, local polarity, or interactions with neighbouring functional groups [41]. This could be triggered by the existence of GNPs.

### 3.5. Ultraviolet-Visible Spectroscopy (UV-Vis)

**Figure 8:**
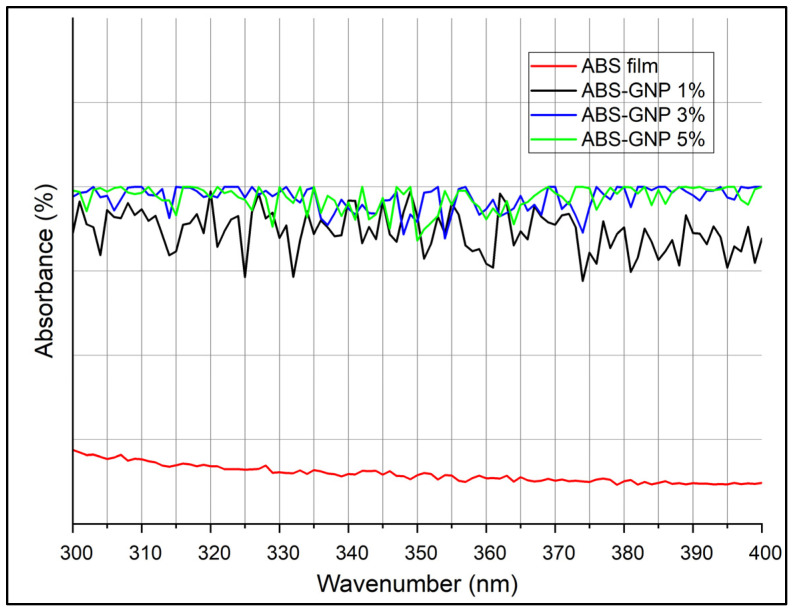
Absorbance spectra of pure ABS and ABS-GNP nanocomposites.

The results of an analysis of the diffuse reflectance spectra of UV-Vis for ABS-GNPs nanocomposites to determine their optical properties are depicted in Figure 8. The enhanced UV radiation shielding capability of ABS-based nanocomposites subsequent to mass-suspension polymerization can be attributed to the incorporation of graphene nanoplatelets (GNPs) into the ABS matrix. A multitude of factors contribute to this phenomenon: The inherent limitations of ABS’s ultraviolet radiation absorption capabilities stem from its chemical composition. However, substantial improvements in ultraviolet (UV) absorption were detected upon integrating graphene nanoplatelets (GNPs) at varying concentrations (1%, 3%, and 5%) into the ABS matrix. UV absorption improved significantly with GNP addition, with 5 wt% samples demonstrating ~55% absorbance at 300 nm, compared to ~18% in pure ABS.

The improvement is also facilitated by the mechanism embodied in the unique attributes of GNPs. Aside from their remarkable optical properties, which include high absorption coefficients, graphene nanoplatelets continue to astound at ultraviolet wavelengths. By virtue of their ability to effectively capture photons within this particular wavelength range, when integrated into the ABS matrix, these GNPs operate as efficient absorbers of UV radiation. Graphene nanoplatelets possess an outstanding electronic structure and a substantial surface area, which capacitate them to effectively absorb ultraviolet radiation. Previous studies have demonstrated that materials composed of graphene possess strong ultraviolet (UV) absorption properties. This is attributed to the formation of electron-hole pairs and the π-electron configuration, which are generated upon exposure to UV radiation [42].

In addition, the enhancement of the nanocomposite in a wavelength-dependent manner is evident as the concentration of GNPs increases. This phenomenon is explained by the increased UV absorption surface area made feasible by the dispersion of more GNPs throughout the ABS matrix. Moreover, through electronic transitions or surface plasmon resonance, optical properties may be enhanced further by the interaction of the GNPs with the ABS polymer matrix. By improving the dispersion of GNPs, mass-suspension polymerization techniques can enhance the particles’ UV protection properties [43]. Rodriguez-Tobas et al. [5] stated that an additional augmentation in ultraviolet absorption might ensue from the synergistic effects produced by the interaction between GNPs and the ABS matrix. As a potential outcome of incorporating GNPs into the nanocomposite, ultraviolet absorption may be enhanced relative to ABS in its pure state. This phenomenon is commonly observed in polymer nanocomposites, where the amalgamation of unique materials yields enhanced performance [38].

The UV absorption characteristics are additionally impacted by the GNP concentration that is incorporated into the ABS matrix. The results of the study indicate that the nanocomposite demonstrates improved ultraviolet (UV) absorption properties as the percentage of GNPs increases from 1% to 5%. Thus, it appears that the relationship between GNP concentration and the efficacy of UV protection is dose-dependent [44].

To summarize, the enhanced ultraviolet protection properties of ABS-based nanocomposites following the incorporation of GNPs and mass-suspension polymerization can be attributed to the following factors: the concentration of GNPs incorporated, the UV absorption capabilities of the GNPs, and their consistent distribution throughout the polymer matrix, as well as the synergistic interactions that occur between the GNPs and ABS.

**Figure 9:**
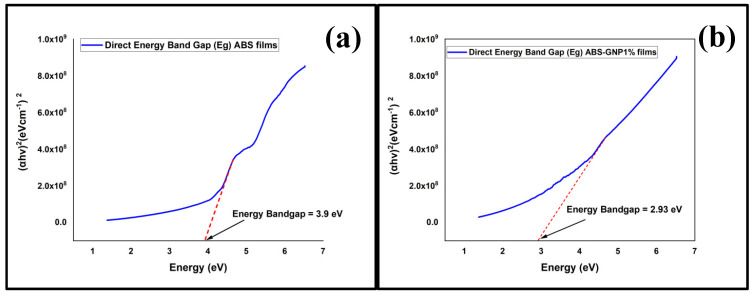

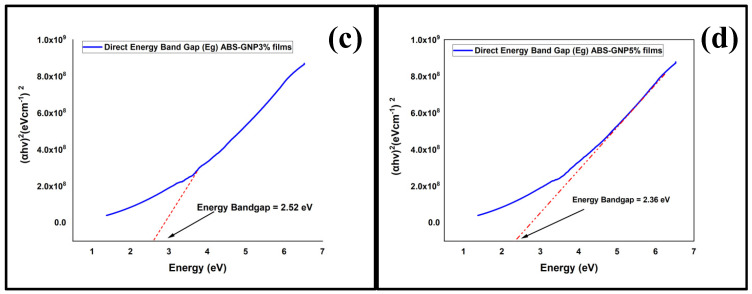
Direct energy optical band gaps for (**a**) pure ABS, (**b**) 1% GNP, (**c**) 3%, and (**d**) 5% ABS-GNP films.

Figure 9 displays the direct energy band gap (DB) for both pure ABS and ABS/GNPs films. The direct energy band gap (DB) is the precise term used to describe the energy difference between the highest energy level in the valence band and the lowest energy level in the conduction band of a material. The inclusion of GNPs modifies the electrical characteristics of the ABS polymer through contact with the polymer matrix. Due to their graphene structure, GNPs have unique electrical properties that might impact the band structure of the polymer. A decrease in the direct energy band gap indicates an enhancement in the conductivity of the material. The observed phenomena can be explained by the inclusion of additional electronic states within the band gap area or a modification in the electronic structure of the polymer. An increasingly noticeable decrease in the direct energy band gap is noticed when the proportion of GNPs increases from 1% to 3% and eventually 5%. This suggests that higher GNP concentrations have a stronger impact on the electrical properties of the polymer. Furthermore, the decrease in the direct energy band gap (DB) of the ABS polymer when GNPs are added can be attributed to numerous factors: (a) The inclusion of GNPs into the polymer matrix, especially at nanoscale dimensions, can cause the occurrence of quantum confinement phenomena. GNPs can reduce the band gap energy by restricting the electrical wavefunctions of the polymer. The occurrence of this phenomenon has been recorded in multiple polymer nanocomposites that contain graphene-based components [45,46]. (b) Charge Transfer: The occurrence of charge transfer can arise from the interaction between the polymer matrix and the graphene nanoplatelets. This charge transfer can modify the electrical structure of the polymer, leading to a decrease in the energy of the band gap. The relationship between the degree of charge transfer and the concentration of GNP can account for the observed tendency of a more significant decrease in DB as the proportion of GNP increases [47,48]. Option (c) Enhanced Interfacial Interaction: Integrating GNPs into the polymer matrix can enhance the interfacial contact between the filler particles. The increased interaction between the materials might cause changes in the arrangement of electrons near the interface, which in turn affects the overall band structure of the fabric [49].

**Figure 10:**
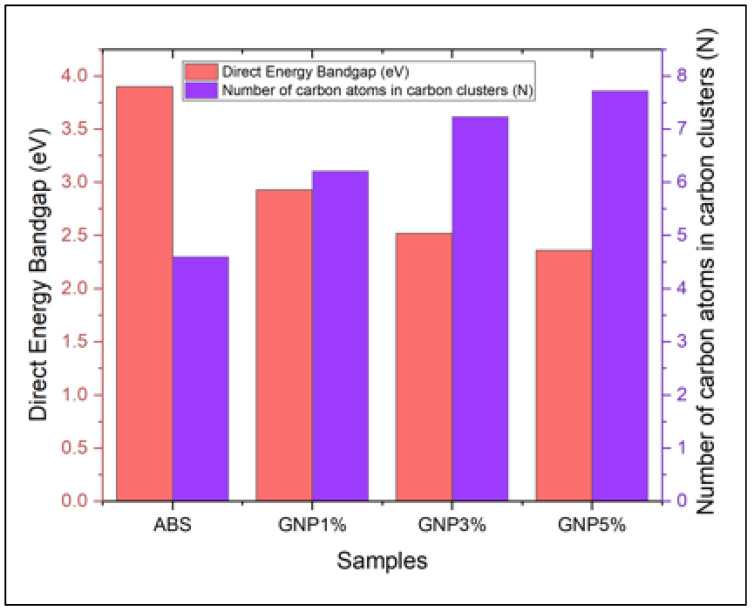
Number of carbon atoms in carbon clusters (N) of ABS-GNPs films.

Regarding the quantity of carbon atom clusters (N), Figure 10 provides an illustration of this. It indicates that a greater number of carbon-rich regions have formed within the polymer matrix, as indicated by the increase in the quantity of carbon atom clusters (N). Attributable to the high aspect ratio and surface properties of the clusters, the dispersion of GNPs within the polymer induces agglomeration and the formation of networks or clusters. The carbon atom clusters within the polymer matrix permit the efficient transport of charge carriers by functioning as conductive pathways. A greater quantity of carbon atom clusters (N) is generated as the proportion of GNPs increases and these particles become more readily available to aggregate within the polymer. Through the enhancement of the polymer’s conductivity, this increase in carbon atom clusters contributes to the observed decrease in the direct energy band gap. Furthermore, the dispersion characteristics and aggregation tendencies of the graphene nanoplatelets (GNPs) can explain the observed increase in the number of carbon atom clusters (N) as the percentage of GNPs in ABS polymer rises: (a) The formation of agglomerations and clusters: At low concentrations, carbon atoms may aggregate into minuscule groupings or agglomerates due to the non-uniform dispersion of GNPs within the polymer matrix. A greater degree of intermingling and proximity among the filler particles [50,51] may cause the clusters to increase in both quantity and amplitude as the concentration of GNPs rises. (b) Percolation threshold: An elevated likelihood of interconnected networks of filler particles forming becomes apparent when the concentration of GNPs surpasses a designated threshold known as the percolation threshold. Due to this, the polymer matrix may undergo the formation of carbon atom clusters of greater size [52]. (c) Interactions between the polymer matrix and the filler particles: The interactions between the polymer matrix and the GNPs have the potential to impact the dispersion behavior of the filler particles [53]. Variations in these interactions resulting from different concentrations of GNP have the potential to impact the propensity of nanocomposite material to aggregate.

Briefly, the incorporation of GNPs into ABS polymer results in an increase in the number of clusters composed of carbon atoms and a decrease in the band gap at the direct energy level. Changes occur due to the interaction between the polymer matrix and graphene nanoparticles (GNPs). These modifications to the electronic structure of the material and the formation of conductive pathways result from this interaction.

### 3.6. Dielectric

**Figure 11:**
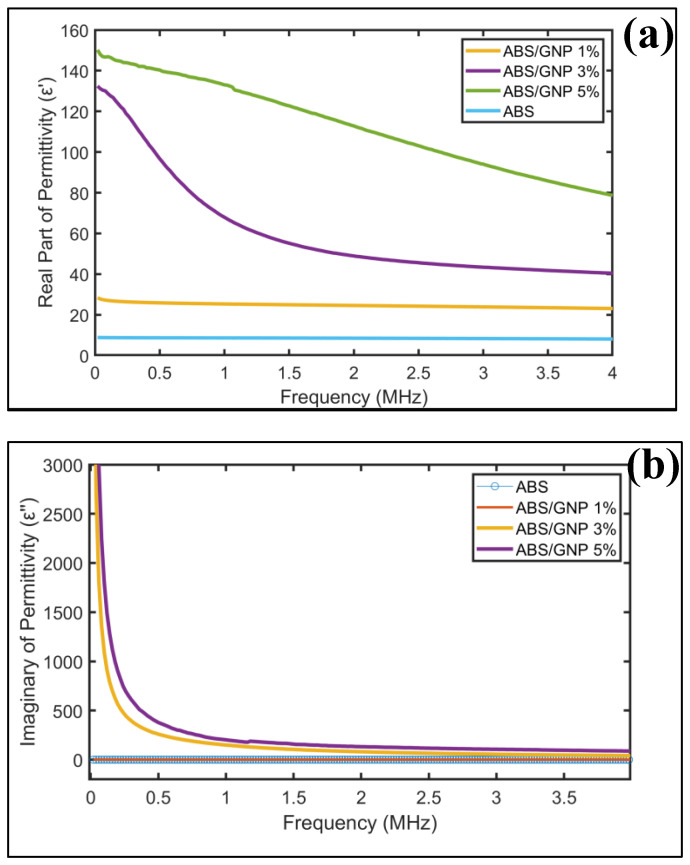
(**a**) The real permittivity of polymer with various GNP concentrations; (**b**) The imaginary permittivity (loss factor) of polymer with various GNP concentrations.

Figure 11a illustrates the real part permittivity of the thin film at different percentages of GNP. In the absence of GNP in the polymer, the ε′ remains relatively consistent [54,55,56] across the entire frequency spectrum. Upon the addition of 1% GNP to the polymer, ε′ rises to above 20, marking the onset of material dispersion and the emergence of exponential characteristics. Increasing the GNP percentage to 3% results in ε′ peaking at 130 at 0.02 MHz, followed by an exponential decline to 40 at 4 MHz. Further increments in GNP percentage continue to elevate ε′, reaching 80 at 4 MHz. The incorporation of the additive alters the non-dispersive nature of the ABS, with ε′ values increasing proportionally to the additive percentage.

In Figure 11b, the imaginary part of the permittivity, commonly referred to as the loss factor, for the thin film is presented. The pure ABS exhibits a minimal loss factor (≈0). With the addition of 1% GNP into the polymer, the loss factor experiences a mild increase up to 1. However, the values escalate significantly in thin films containing 3% and 5% of GNP. At 0.1 MHz, polymers containing 3% and 5% of GNP exhibit loss factors of 1090 and 1800, respectively. Particularly below 0.5 MHz, the exponential nature of the loss factor becomes pronounced in polymers containing 3% and 5% of GNP. Subsequently, the values decrease to 40 (3% GNP) and 90 (5% GNP) at 4 MHz. The addition of the GNP into the polymer has enhanced the conductivity of the polymer as the relationship between the loss factor and conductivity is described by Equation (1).

### 3.7. EMI Shielding

**Figure 12:**
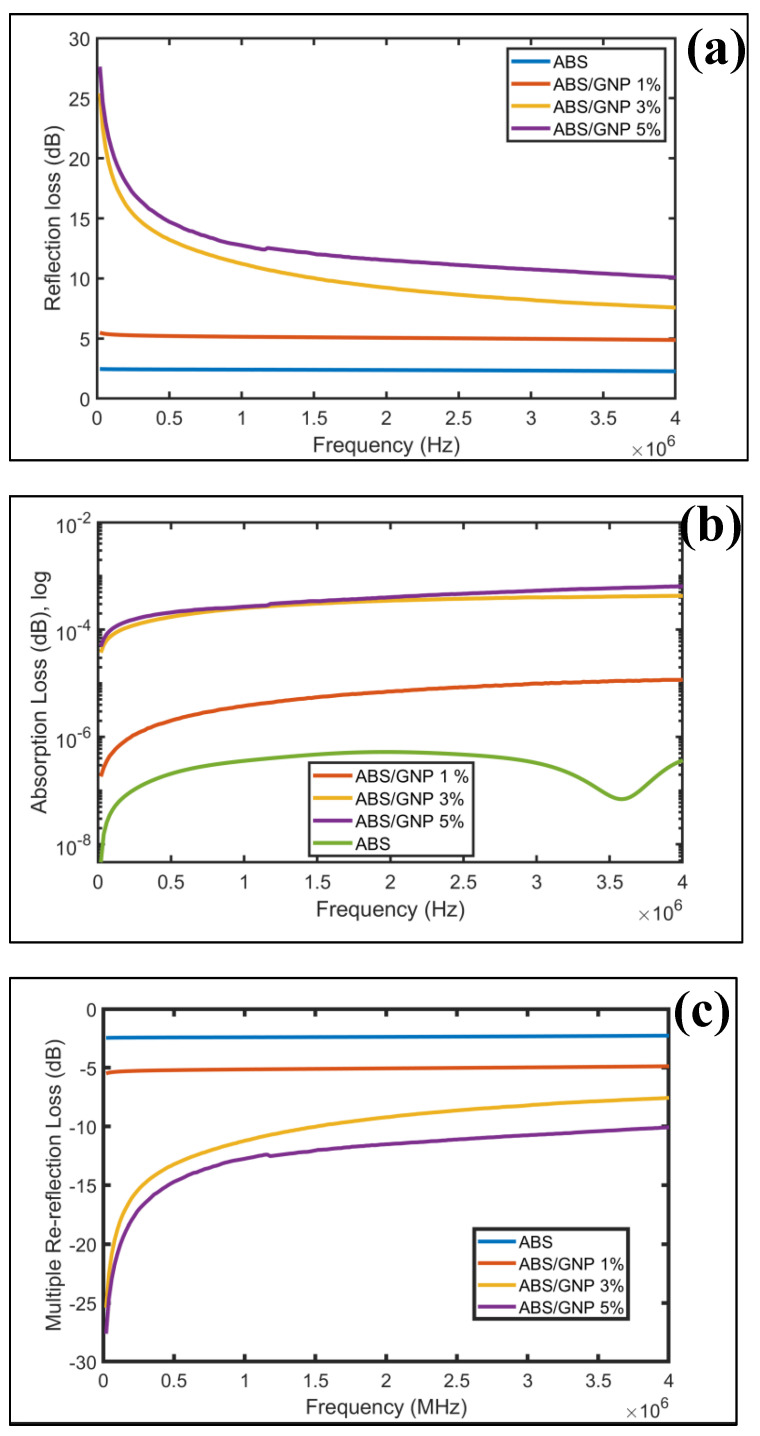

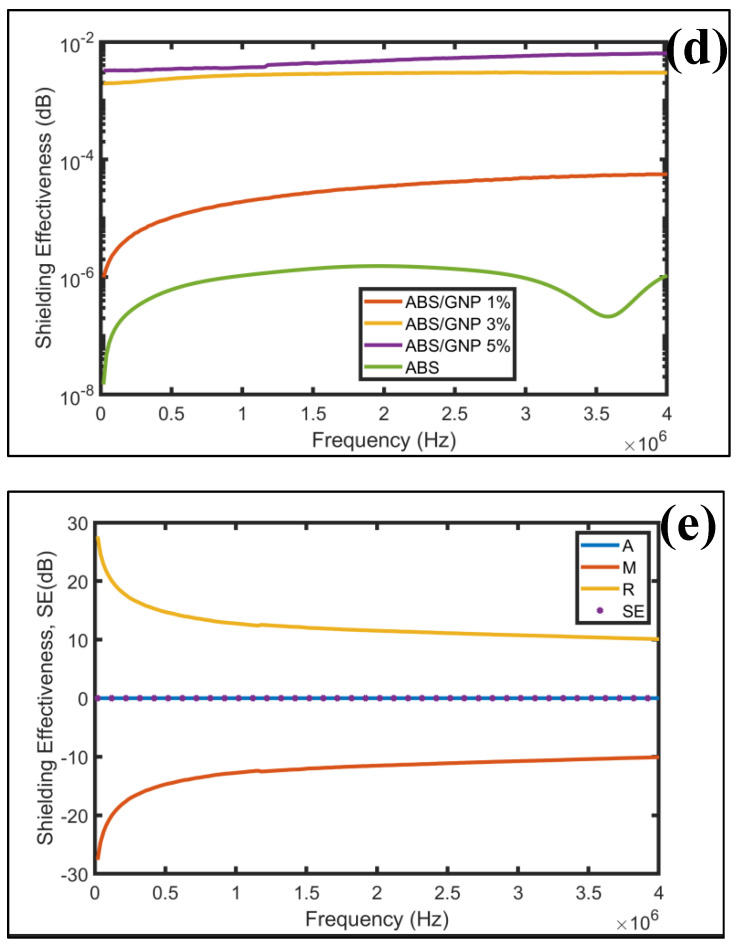
(**a**) Reflection loss (dB) for polymer samples with varying graphene nanoplatelet (GNPs) percentages, (**b**) Absorption loss (dB) in logarithmic scale for polymer samples with varying graphene nanoplatelet (GNPs) percentages, (**c**) Multiple re-reflection loss (dB) for polymer samples with varying graphene nanoplatelets (GNPs) percentages, (**d**) Shielding effectiveness (dB) in logarithmic scale for polymer samples with varying graphene nanoplatelets (GNPs) Percentages, (**e**) The comparison of SE, R, A, and M of ABS/GNPs at 5% percentage.

The integration of graphene nanoplatelets (GNPs) as conductive fillers within the acrylonitrile butadiene styrene (ABS) matrix significantly improves the electromagnetic (EM) wave attenuation capability of the polymer, primarily by mitigating impedance mismatches. As depicted in Figure 12a, the pristine ABS polymer exhibits relatively low reflection loss, with a maximum value of approximately 2.5 dB across frequencies up to 4 MHz. Upon the incorporation of 1 wt% GNPs, a marked enhancement in reflection loss is observed, reaching up to 5 dB consistently across the entire measured frequency range. Further increases in GNP loading to 3 wt% and 5 wt% yield even more pronounced improvements, particularly at frequencies below 1 MHz. At this lower frequency range, the reflection loss peaks at 12 dB and 13 dB for 3 wt% and 5 wt% GNP-filled composites, respectively. A reflection loss of 13 dB corresponds to approximately 22% reflection of the incident EM wave, indicating a substantial enhancement in the material’s shielding performance. These findings highlight the efficacy of GNPs in tuning the electromagnetic interference (EMI) shielding characteristics of polymer-based composites.

The study of absorption loss in acrylonitrile butadiene styrene (ABS) composites reinforced with varying concentrations of graphene nanoplatelets (GNP), as illustrated in Figure 12b, reveals that the attenuation of electromagnetic (EM) waves within the material is minimal across all tested formulations. Even at a GNP content of 5 wt%, the measured absorption loss is only 6.5 × 10⁻^4^ dB, indicating that the filler does not significantly contribute to EM wave attenuation.

This limited absorption is primarily attributed to two factors: the non-magnetic nature of the ABS/GNP composite and the small thickness of the sample, which is just 0.2 mm. Due to the absence of magnetic components, the composite exhibits a high skin depth, as described by Equation (6). Consequently, achieving a substantial reduction in wave intensity (to below 1/e) would require a thicker material. Moreover, because the analysis was conducted in the low-frequency range, absorption becomes even more challenging, since magnetic and dielectric losses are less effective at such frequencies.

In comparison, the behavior of multiple re-reflection loss—depicted in Figure 12c—presents a different trend. Notably, higher GNP content leads to a decrease in multiple re-reflection loss. Among the tested samples, the 5 wt% GNP composite shows the lowest re-reflection loss, followed by the 3 wt% and 1 wt% GNP formulations. Interestingly, the pure ABS sample (0% GNP) demonstrates the highest re-reflection loss.

This pattern can be explained by the thin nature of the ABS films, which limits their capacity for absorption. As a result, a significant portion of the incident EM waves undergoes internal reflections. In composites with lower GNP concentrations or none at all, these reflections are more frequent and may contribute to reinforcing rather than attenuating the waves. This highlights a unique electromagnetic behavior where the interaction between GNP content, material thickness, and wave propagation plays a critical role.

The cumulative impact of reflection, absorption, and re-reflection losses is reflected in the overall shielding effectiveness (SE), as presented in Figure 12d,e. The highest SE value observed is 0.0064 dB at 4 MHz, achieved by the composite containing 5 wt% GNP. Although this value is the best among the samples, it remains low for practical EMI shielding applications.

Several key factors contribute to this low SE: the ultra-thin film thickness leads to a high skin depth, while the lack of magnetic fillers reduces absorption potential. Additionally, testing at low frequencies further limits shielding efficiency, as stronger interactions and losses are typically observed at higher frequencies.

Despite the high conductivity and permittivity introduced by GNPs—which enhance reflection loss—the overall SE is diminished due to compensation by the multiple re-reflection loss. This suggests a balancing effect between the mechanisms, where internal reflections limit the net shielding capability.

To improve the SE performance of thin-film ABS-based composites, the following strategies are recommended: 1-Incorporate magnetic nanoparticles (e.g., Fe_3_O_4_) to enhance magnetic permeability, thereby reducing skin depth and increasing absorption, 2-Design multilayer or foam structures to extend the interaction length of EM waves within the material, 3-Utilize hybrid filler systems, such as combinations of GNP with carbon nanotubes (CNTs) or ferrites, to improve both dielectric and magnetic losses.

### 3.8. Field Emission Scanning Microscopy (FESEM)

**Figure 13:**
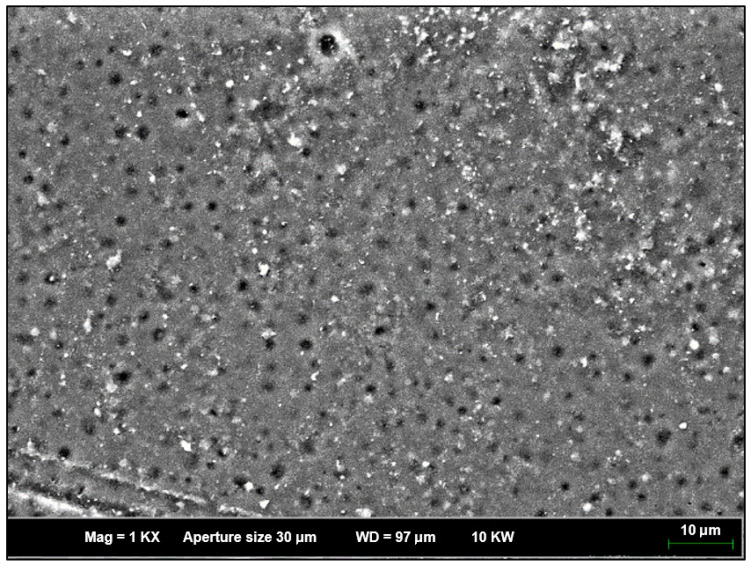
FESEM image of pure acrylonitrile butadiene styrene (ABS) film showing a porous microstructure with granular surface features.

**Figure 14:**
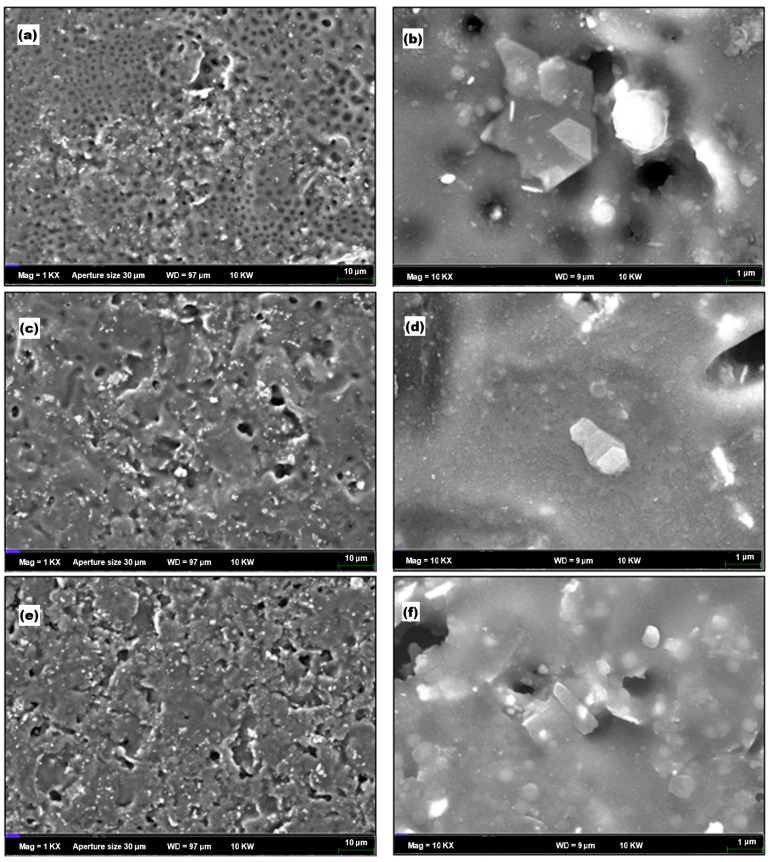
FESEM images of nanocomposites (**a**) 1 wt% GNPs at 1 k magnification, (**b**) 1 wt% GNPs at 10 k magnification, (**c**) 3 wt% GNPs at 1 k magnification, (**d**) 3 wt% GNPs at 10 k magnification, (**e**) 5 wt% GNPs at 1 k magnification and (**f**) 5 wt% GNPs at 10 k magnification.

The morphological features of pure acrylonitrile butadiene styrene (ABS) and graphene nanoplatelet-reinforced ABS (ABS/GNPs) nanocomposites were investigated using Field Emission Scanning Electron Microscopy (FESEM). All samples were sputter-coated with a thin layer of platinum prior to imaging to improve surface conductivity. The FESEM analysis aimed to assess the dispersion of GNPs, filler-matrix compatibility, and interfacial connectivity between the polymer and the nanofiller. Representative FESEM images of both pure ABS and ABS/GNPs composite films are presented in Figure 13 and Figure 14.

As shown in Figure 13, the pure ABS film exhibits a porous microstructure with granular surface features, which is consistent with previous findings [2,3,6,7]. This morphology can be attributed to the intrinsic phase-separated nature of ABS, where polybutadiene (PB) domains are embedded within a continuous styrene-acrylonitrile (SAN) matrix [57]. These phase domains contribute to the formation of pores and heterogeneous textures in the polymer matrix.

The incorporation of GNPs into the ABS matrix induces notable morphological changes, as illustrated in Figure 14. GNPs effectively fill the voids present in the pure ABS film, indicating improved blend compatibility and interfacial adhesion [58]. Their two-dimensional structure allows them to penetrate and interact with the polymer network, enhancing the microstructural integrity of the composite. The improved interface between GNPs and ABS contributes to enhancements in stiffness, tensile strength, and overall mechanical performance [59].

At lower magnifications (1 k), the nanocomposite surface reveals dispersed granule-like features corresponding to uniformly distributed GNPs [6]. Upon increasing the magnification to 10 k, the films exhibit semi-crystalline regions, indicating localized ordering induced by GNP incorporation [60]. These crystalline domains can contribute to improvements in thermal and barrier properties.

Moreover, FESEM images of ABS/GNP composites containing 1–3 wt% GNPs (Figure 14a–d) reveal hexagonal and layered graphene structures, consistent with previously reported morphologies of graphene-based nanocomposites [61]. The characteristic folded and wrinkled sheet-like structures observed at higher magnifications (~10,000×) further support the presence of exfoliated GNPs within the polymer matrix [50]. These morphological traits are crucial for enhancing mechanical reinforcement and EMI shielding effectiveness.

A uniform dispersion of GNPs and strong interfacial adhesion is observed up to 3 wt% GNP loading (Figure 14d), suggesting successful matrix-filler integration [62]. However, at higher GNP content (5 wt%), visible agglomeration occurs (Figure 14e), potentially leading to localized stress points and diminished mechanical or electrical properties [63]. The observed rectangular agglomerates under 10 k magnification (Figure 14f) likely represent stacked or clustered GNPs that reduce the overall homogeneity of the nanocomposite.

In summary, the morphological analysis demonstrates that GNP addition significantly alters the microstructure of ABS. The improved dispersion, reduced porosity, and enhanced interfacial interactions underscore the role of GNPs in tailoring polymer properties. These findings are consistent with prior studies on functional nanocomposites and further support the potential of GNP-reinforced ABS for high-performance applications [2,3,6,7].

## 4. Conclusions

This study successfully demonstrated the fabrication and comprehensive characterization of graphene nanoplatelet (GNP)-reinforced acrylonitrile butadiene styrene (ABS) nanocomposite films via a sonication-assisted casting method. The incorporation of GNPs into the ABS matrix was confirmed through a suite of material characterization techniques, which collectively indicated improved dispersion and minimal agglomeration—addressing key challenges in nanofiller integration. Field emission scanning electron microscopy (FESEM) revealed a homogeneous microstructural distribution, while thermal analysis via TGA and DSC showed enhanced thermal stability and elevated decomposition temperatures in the GNP composites, attributable to the thermal barrier properties and intrinsic conductivity of GNPs. Dielectric assessments further demonstrated a reduction in both dielectric constant and loss tangent upon GNP addition, suggesting the material’s suitability for low-dielectric electronic applications. Significantly, electromagnetic interference (EMI) shielding tests indicated that GNP-filled ABS films achieved superior shielding effectiveness, driven by enhanced electromagnetic absorption and dissipation resulting from the conductive GNP network. These findings highlight the promising multifunctionality of GNP-reinforced ABS nanocomposites, positioning them as strong candidates for applications in automotive, aerospace, and electronic sectors where enhanced thermal, dielectric, and EMI shielding characteristics are critically required. This work underscores the potential of tailoring polymer nanocomposites for high-performance applications through scalable and efficient processing strategies.

## Figures and Tables

**Table 1:** Formulation of GNP-reinforced ABS nanocomposite films.

Sample	Formulation (wt%)	
	GNPs	ABS
Control sample	0	100
ABS-GNP 1	1	99
ABS-GNP 3	3	97
ABS-GNP 5	5	95

**Table 2:** The summary of water contact angle and surface energy of ABS and its nanocomposites.

Samples	Contact Angle (°)	Surface Energy (mN/m)
pure ABS	74.14	39.87
ABS-GNP 1%	66.54	44.12
ABS-GNP 3%	88.76	30.14
ABS-GNP 5%	92.61	27.63

**Table 3:** Summary of TGA curves at different temperatures.

Samples	T_20_ (°C)	T_50_ (°C)	T_80_ (°C)
Pure ABS	345	396.67	472.53
ABS-GNP 1%	354.17	402.47	431.69
ABS-GNP 3%	372.50	407.51	432.51
ABS-GNP 5%	362.47	397.47	422.47

**Table 4:** Summary of DSC curves for ABS-GNP polymer nanocomposites.

Samples	T_g_ (°C)
Pure ABS	109.82
ABS-GNP 1%	110.75
ABS-GNP 3%	109.94
ABS-GNP 5%	123.81

## Data Availability

The original contributions presented in this study are included in the article. Further inquiries can be directed to the corresponding author.
